# A Novel Time–Frequency Parameterization Method for Oscillations in Specific Frequency Bands and Its Application on OPM-MEG

**DOI:** 10.3390/bioengineering11080773

**Published:** 2024-07-31

**Authors:** Xiaoyu Liang, Ruonan Wang, Huanqi Wu, Yuyu Ma, Changzeng Liu, Yang Gao, Dexin Yu, Xiaolin Ning

**Affiliations:** 1 School of Instrumentation Science and Optoelectronic Engineering, Beihang University, Beijing 100191, China; liangxiaoyu17@buaa.edu.cn (X.L.); by1917076@buaa.edu.cn (R.W.); whqmeg@buaa.edu.cn (H.W.); ma_yuyu@buaa.edu.cn (Y.M.); liu674491721@163.com (C.L.); yanggao@buaa.edu.cn (Y.G.); 2 Key Laboratory of Ultra-Weak Magnetic Field Measurement Technology, Ministry of Education, School of Instrumentation and Optoelectronic Engineering, Beihang University, Beijing 100191, China; 3 Hefei National Laboratory, Hefei 230088, China; 4 Institute of Large-Scale Scientific Facility and Centre for Zero Magnetic Field Science, Beihang University, Hangzhou 310051, China; 5 National Institute of Extremely-Weak Magnetic Field Infrastructure, Hangzhou 310051, China; 6 Shandong Key Laboratory: Magnetic Field-Free Medicine & Functional Imaging, Qilu Hospital of Shandong University, Jinan 250012, China; ydx0330@sina.com

**Keywords:** MEG, cortical oscillations, time–frequency parameterization, aperiodic component fitting

## Abstract

Time–frequency parameterization for oscillations in specific frequency bands reflects the dynamic changes in the brain. It is related to cognitive behavior and diseases and has received significant attention in neuroscience. However, many studies do not consider the impact of the aperiodic noise and neural activity, including their time-varying fluctuations. Some studies are limited by the low resolution of the time–frequency spectrum and parameter-solved operation. Therefore, this paper proposes super-resolution time–frequency periodic parameterization of (transient) oscillation (STPPTO). STPPTO obtains a super-resolution time–frequency spectrum with Superlet transform. Then, the time–frequency representation of oscillations is obtained by removing the aperiodic component fitted in a time-resolved way. Finally, the definition of transient events is used to parameterize oscillations. The performance of this method is validated on simulated data and its reliability is demonstrated on magnetoencephalography. We show how it can be used to explore and analyze oscillatory activity under rhythmic stimulation.

## 1. Introduction

Neural oscillations have been extensively studied over the past century. Studies have shown that some oscillations in specific frequency bands reflect various cognitive, perceptual, and behavioral states [1,2] and contribute to coordinating information transfer between brain regions [3,4]. For instance, the theta-band (4–7 Hz) oscillation is related to tasks such as memory, navigation, and cognitive processing [5,6]. The alpha-band (7–12 Hz) oscillation is related to aging [7], development [8], temporal organization of perception [9], and so on. The beta-band (12–30 Hz) oscillation plays a role in neurodevelopmental trajectory [10], movement [11], and so on. The parameters of these specific frequency band oscillations, such as frequency, frequency span, onset time, and duration, vary with changes in behavior and physiological processes, reflecting the dynamic states and networks of the brain [4,12,13,14]. Generally, time-domain neural signals are transformed into a frequency spectrum or time–frequency spectrum by time–frequency analysis. Oscillatory activities are defined as peaks in the spectrum. Thus, these parameters can be computed from the spectrum. For example, these parameters can be obtained by defining oscillations as the peak power above a cut-off value of 6× the median power of the time–frequency [15] or via Hidden Markov Modeling (HMM) [16].

However, aperiodic noises, such as single impulse functions and white noise, affect the analysis of oscillations in practice. Additionally, neural signals also contain aperiodic activities. The power of these aperiodic components is always non-zero [17,18]. The above techniques may mistake any changes in aperiodic components for changes in oscillation parameters. Therefore, it is necessary to separate oscillatory and aperiodic components in the frequency spectrum or time–frequency spectrum to accurately determines the parameters of oscillations. This challenge led to the development of the ‘fooof’ (fitting oscillation and one over f) algorithm [7], which parameterizes the oscillatory and aperiodic components from the frequency spectrum. This method has been widely used in studies of brain aging [19], development [20], and disease [21,22]. However, it parameterizes oscillations from a static spectrum, which does not contain temporal information. Brady and Bardouille proposed PAPTO to obtain temporal and frequency information of oscillations simultaneously, such as oscillation frequency span and duration [23]. The time–frequency representation (TFR) of oscillations is normalized by the modeled aperiodic component power using ‘fooof’. It reduces the impact of the aperiodic component on the parametrizing oscillations by fitting the aperiodic component from the static spectrum and removing it. This operation assumes that the aperiodic component remains constant throughout the entire data. However, several studies have demonstrated changes in the aperiodic component over time [24,25]. This aspect is not considered in the above. As a result, it may introduce false oscillation components. For example, higher power at frequencies where the actual oscillatory power is low (see Figure S2). This leads to inaccurate TFR of oscillations separated from the aperiodic background, resulting in inaccurate oscillation parameterization. Therefore, when separating oscillation from an aperiodic background, it is necessary to consider that the aperiodic component also varies with time [20]. For the time-varying aperiodic component, Wilson et al. proposed Spectral Parameterization Resolved in Time (SPRiNT) [26]. It solves the time–frequency spectrum using Short-time Fourier Transform (STFT). Then, it uses ‘fooof’ to fit oscillations with Gaussian functions in each time window. Finally, the peak frequency and time of the oscillations can be obtained [26]. However, it does not calculate some parameters such as frequency span and duration. Meanwhile, it calculates the time–frequency spectrum using STFT, which is significantly influenced by the Heisenberg–Gabor uncertainty principle, leading to limited time–frequency resolution. For this problem, the Superlet Transform (SLT) proposed by Moca can effectively improve it [27]. Furthermore, it offers a super time–frequency resolution compared to the wavelet transform utilized in PAPTO [23] and other conventional time–frequency analysis methods.

Therefore, this paper proposes the super-resolution time–frequency periodic parameterization of (transient) oscillation (STPPTO) as a novel method for improved time–frequency parameterization of oscillations. Firstly, it obtains the super-resolution time–frequency spectrum with SLT. Then, the TFR of the aperiodic component is calculated in a time-resolved manner. The TFR of oscillations is obtained by removing aperiodic components from the time–frequency spectrum. This operation reduces the impact of the time-varying aperiodic component on the analysis of oscillations in specific frequency bands. Finally, the definition of transient events [9,14] is used to calculate the oscillation parameters from the TFR of the oscillations, such as onset time, peak frequency, duration, and frequency span. The objectives of this paper are (1) to motivate and validate the accuracy of the proposed algorithm compared to previous methods by simulations, (2) to quantify the comparison between algorithms in simulations, superconducting quantum interference device-based magnetoencephalography (MEG) (SQUID-MEG) and optically pumped magnetometer (OPM)-based MEG (OPM-MEG), (3) validate that there are slight differences between SQUID-MEG and OPM-MEG in the oscillatory parameters solved by STTPTO, and (4) to validate the reliability of STPPTO and apply it on multi-trial flash stimuli (FS) measured by OPM-MEG.

The main points of the article are as follows. Firstly, the performance of STPPTO is tested with simulated neural signals. It demonstrates that the algorithm can compute peak frequency, frequency span, onset time, and duration of oscillations with fewer errors compared to existing methods. Subsequently, we apply STPPTO to analyze oscillations of the primary visual cortex in resting SQUID-MEG and OPM-MEG, accompanied by quantitative comparisons against existing algorithms. We demonstrate that some oscillatory parameters are close between different MEG techniques. Finally, we calculate the coefficient of variation in multi-trial oscillation parameters to verify the multi-trial reliability of the method under FS OPM-MEG data. Furthermore, we apply the proposed method to analyze oscillation responses between different-order harmonics and dominant hemispheres.

## 2. Materials and Methods

### 2.1. Human Subjects

Resting-state SQUID-MEG recordings are available for 48 (age: mean = 21.3, range = 18–29, gender: 31 females) out of a total of 204 healthy participants of the MOUS dataset [28]. All subjects were right-handed, had normal or corrected-to-normal vision, and reported no history of neurological, developmental, or language deficits. The MOUS study was approved by the local ethics committee (CMO—the local “Committee on Research Involving Human Subjects” in the Arnhem-Nijmegen region) and followed the guidelines of the Helsinki declaration. 

OPM-MEG rest-state and FS data are available for 9 healthy subjects (age: mean = 27.7, range = 25–29, gender: 2 females). All subjects were right-handed, had normal or corrected-to-normal vision, and reported no history of neurological, developmental, or language deficits. The project was reviewed and approved by the Biomedical Ethics Committee of Beihang University. All ethical regulations related to human experimentation, including the “Declaration of Helsinki”, are complied. The experiment and photos were used with the consent and permission of the subjects. 

### 2.2. Simulation and MEG Experiment

#### 2.2.1. Stimulated Neural Data

- Simulation I: Simulated neural data consist of aperiodic and oscillatory activity

In Simulation I, a single trial lasts 2 s, and 200 epochs are simulated. The parameters of the simulated oscillation signals include theta, alpha, beta, and gamma band oscillations (the specific values as shown in Table S1 in the Supplement). For the aperiodic component, the exponent and offset are set to 1.5 and −2.56 during the first 1.2 s and −1.44 and 1.75 during the last 0.8 s. The neural data algorithm is based on NeuroDSP [29] (https://github.com/neurodsp-tools/neurodsp (accessed on 31 December 2023)). This simulation is used to evaluate the performance of algorithms in parameterizing oscillations from aperiodic backgrounds.

- Simulation II: Simulated neural data with empty-room noise in OPM-MEG

To better simulate brain activity recorded by MEG with real noise, we combined empty-room noise and simulated neural activity. MRI and co-registration data from one subject (male, 28 years old) were used to construct simulations. The empty-room noise is recorded without a subject in a magnetically shielded room (MSR) in the OPM-MEG experiment. It was collected by 31 OPM sensors (QZFM, QuSpin Inc., Louisville, USA) distributed on the helmet, which covers an occipital lobe if the subject wears it. And, empty-room noise was preprocessed (bad channel removing (26 channels left), filtered, and corrected by homogenous field correction (HFC) [30] similar to the following OPM-MEG experiment. Then, the source reconstruction was used to simulate the activation noise caused by the empty room noise in the regions of interest (ROI). The noisy activation was used as the noise signal in ROIs (Calcarine sulcus and Cuneus). Finally, the noise signal was added to the signal in Simulation I as the simulated source time series. This simulation is used to evaluate the performance of algorithms in parameterizing oscillations from an aperiodic background and complex environmental noise.

#### 2.2.2. Resting-State SQUID-MEG and OPM-MEG

In this study, we used the MOUS dataset [28] (https://data.donders.ru.nl/collections/di/dccn/DSC_3011020.09_236?1 (accessed on 20 December 2023)), which contains five minutes of resting-state MEG recordings. The participants were instructed to think of nothing specific with their eyes open. Data were collected using a CTF 275-channel SQUID-MEG system. The anatomical images of the head were obtained with a SIEMENS Trio 3T (volume TR = 2300 ms, TE = 3.03 ms, eight-degree flip-angle, one slab, slice-matrix size = 256 × 256, slice thickness = 1 mm, field of view = 256 mm, isotropic voxel size = 1.0 × 1.0 × 1.0 mm). 

The resting-state OPM-MEG experiment contained five minutes of resting-state MEG recordings. The MEG was recorded by 31 dual-axis OPM sensors (QZFM, QuSpin Inc., Louisville, USA). A 3D-printed rigid helmet was used with slots placed to hold the sensors according to a 10–20 system layout. In total, 31 sensors covered the occipital lobe region, evenly distributed on the left and right sides. The OPM sensors were configured to measure the radial components of the magnetic field with a single axis, which enabled 31-channel data collection. In a magnetic shield room (MSR) (ambient field amplitude and drift typically below 13 nT and 40 pT/h), MEG was recorded by a PXI computer chassis (PXIC-7318C, ART Technology Inc., Beijing, China). The anatomical images of the head were obtained with a Siemens MAGNETOM Prisma 3T MR system. T1-weighted MRI scans were obtained with an MPRAGE sequence (TR, 2300 ms; TE, 3.03 ms; TI, 1100 ms; FA, 8; field of view, 256 × 256 ×192 mm; voxel size = 1.0 × 1.0 × 1.0 mm). MRI data were pre-processed and segmented with Freesurfer (Version 6) software [31] to obtain the scalp surface and cortex.

#### 2.2.3. OPM-MEG under Rhythmic Flash Stimulus (FS)

The FS experiment paradigm was run as follows. 3 Hz, 6.5 Hz, and 8.5 Hz flash were pseudo-randomly presented in the same proportion in one block. Two blocks of stimulation were applied to the left eye and two blocks to the right eye and each block consisted of 105 trials. Thus, there were 70 trials of each frequency stimulus for each eye. Each trial lasted 2 s, with a 2-s interval between the trials. The flash was introduced into the shielded room by a fiber optic, with a light intensity of 292 lux in the room. Subjects focused on a fiber optic lamp with a unilateral eye (the non-stimulated eye was covered with a medical eye patch) at 40–50 cm. MEG and stimulus triggers were recorded synchronously by a PXI computer chassis (PXIC-7318C, ART Technology Inc., Beijing, China). The other experimental equipment, operation, and MRI data were the same as those in the resting-state OPM-MEG experiment. 

#### 2.2.4. MEG Data Preprocessing

The resting-state SQUID-MEG data were band-pass filtered from 1–46 Hz, with notch filtering at 50 Hz. Then, independent component analysis (ICA) was carried on it (30 components obtained with the infomax method [32], with an average of 11.3 components including artifacts such as eye-movement heartbeats removed (range: 5–14)). After removing 6 subjects with abnormal preprocessing or source reconstruction, we retained 42 subjects for analysis.

Similarly, 240 s of OPM-MEG resting-state data were retained. Subsequently, the resting-state data and the OPM-MEG data under flash stimuli were preprocessed by band-pass filtering from 1–46 Hz, notch filtering at 50 Hz, bad-channel removing (21–26 channels remained after removing the unstable channels), HFC (aiming at OPM-MEG), and ICA (20 components obtained, with an average of 5.6 components removed (range: 3–6)). Then, we retained an average of 62.2 ± 1 epochs out of a total of 70 trials for each stimulus after removing bad trials (with amplitude greater than 2000 ft).

#### 2.2.5. Source Time Series Reconstruction

A 3D model of every subject wearing a helmet was scanned with an infrared light scanner (EinScan H, SHINING 3D Inc., Hangzhou, China) before the experiment to obtain the positions of the sensors relative to the scalp surface [33,34] in the OPM-MEG experiment. Dynamic Statistical Parametric Mapping (dSPM) was used for source time series reconstruction, which combines information from different imaging patterns with a priori anatomical and physiological information to generate spatiotemporal estimates of brain activity that can be accurate to the millisecond [35]. The inverse problem-solving algorithm (loose = 0.2 for the loose constraint factor and depth = 0.8 for the depth weight) and the source localization algorithm in MNE-python [36] (dSPM, the regularization parameter is set to 1/9) were used to reconstruct source signals of ROIs. With “aparc.a2009s” brain template [30], we automatically partitioned the cerebral cortex of each participant and selected ROIs. The source signals of the visual primary regions were reconstructed, including Calcarine sulcus and Cuneus related primary visual processing [37], including S_calcarine-lh (Left V1), S_calcarine-rh (Right V1), G_cuneus-lh (Left O6), and G_cuneus-rh (Right O6) labels in the “aparc.a2009s” template [38].

### 2.3. The Principle of STPPTO

**Figure 1:**
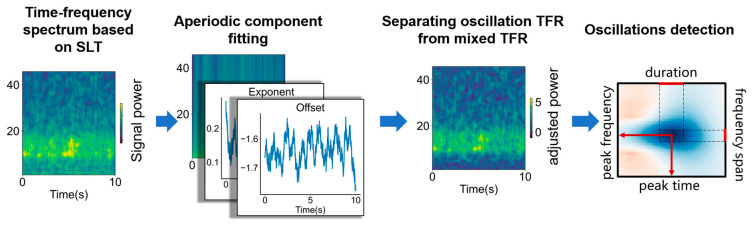
The principle of STPPTO. The process of STPPTO includes calculation of the time–frequency spectrum of source signal with SLT. Then, the TFR of aperiodic components are fitted and periodic TFR are separated. Finally, oscillations from periodic TFR are parameterized.

As show in Figure 1, the time–frequency parameterization process of oscillation activity based on STPPTO includes using SLT to calculate the time–frequency spectrum of source signal, separating TFR of the oscillation (fitting the TFR of aperiodic components, obtaining the time–frequency distribution of the oscillation), and oscillation detection.

#### 2.3.1. Time–Frequency Spectrum of Signal

Superlets is defined as a set of wavelets with a fixed central frequency but varying periods. This enhances the resolution of high-frequency components with longer wavelets, while preserving the excellent temporal resolution of short wavelets of SLT [27]. It obtains a time–frequency spectrum with super-resolution, improving the accuracy of subsequent oscillation analysis. The time–frequency spectrum (in linear space) with a mixture of aperiodic and oscillatory components can be expressed as

(1) $$R_{\mathrm{mix}}\left(t,f\right)=\prod_{i=1}^{o}{\left|\sqrt{2}\frac{1}{a}\int_{-\infty }^{+\infty }x\left(\tau \right)\psi_{i}^{*}\left(\frac{\tau -t}{a}\right)d\tau \right|}^{\frac{1}{o}}$$

(2) a=wmwa

(3) $$\psi_{i}\left(u\right)=\frac{5}{c_{1}\cdot i{\left(2\pi \right)}^{3/2}}e^{-\frac{1}{2}{\left[\frac{5u}{2\pi c_{1}\cdot i}\right]}^{2}}e^{ju},\left(i=1,2,\cdots ,o\right)$$

where $w_{m}$ is the center frequency of the mother wavelet. $w_{a}$ is the frequency to be analyzed. $w_{a}=2\pi f/f_{s}$. $f_{s}$ is the sampling frequency. $\psi_{i}^{*}\left(u\right)$ denotes the conjugation of $\psi_{i}\left(u\right)$. *c*_1_ is the initial value of the superlets.

*o* is the order that determines the number of wavelets in superlets set, which is related to the frequency to be analyzed.

(4) $$o\left(f\right)=o_{\min}+\left[\left(o_{\max}-o_{\min}\right)\cdot \frac{f-f_{\min}}{f_{\max}-f_{\min}}\right]$$

where $o_{\min}$ is the minimum order, take 1 here. $o_{\max}$ is the maximum order, take 30 here. f is the frequency that needs to be analyzed. $f_{\min}$ is the minimum frequency that needs to be analyzed. $f_{\max}$ is the maximum frequency that needs to be analyzed.

#### 2.3.2. Separating Oscillation TFR

The fitting algorithm for TFR of aperiodic components $E_{\mathrm{ap}}\left(t,f\right)$ is adapted from ‘fooof’ (available at https://’fooof’-tools.githu(b)io/’fooof’/index.html (accessed on 20 October 2023)) [7]. In STPPTO, the frequency–power distribution at each moment is fitted by ‘fooof’. The every-moment fitting result is taken as the initial value for the next moment fitting. The aperiodic TFR fitting implementation flow is shown in Figure S1(a) in Supplement. It is implemented based on MNE-python [36]. Every ‘fooof’ modeling was performed across the entire 1–46 Hz (according to the filter of MEG in practice) spectrum with the peak width limited between 2 and 10 Hz, a minimum peak height of 0.05× (maximum of calculated TFR), a peak threshold of 1.5, and a maximum of 4 peaks.

The power spectrum of a single-trial time series can be modeled as the sum of aperiodic component and oscillation activity [7]. In practice, the aperiodic component and oscillation activity are constantly changing over time. The time–frequency spectrum of single-trial time series can be more finely represented as the sum of the time–frequency fluctuations in the aperiodic background $E_{\mathrm{ap}}\left(t,f\right)$ and oscillation activity $E_{p}\left(t,f\right)$ in the log space [7,23,26]. Therefore, the relationship between the above activities can be modeled as in the log scale

(5) $$\log R_{\mathrm{mix}}\left(t,f\right)=E_{\mathrm{ap}}\left(t,f\right)+E_{p}\left(t,f\right)$$

The mathematical model of aperiodic component can be defined as [13,21,34]

(6) $$E_{\mathrm{ap}}\left(t,f\right)=b_{t}-\log_{10}\left(k+f^{\chi_{t}}\right)$$

where $b_{t\;}$ and $\chi_{t}$ denote the offset and exponent of aperiodic exponents at time t, respectively. k is a knee, k=0 in the general analysis. 

For the oscillatory component, when detecting the local time–frequency events of oscillations in TFR, all peaks in the local oscillation TFR can be defined as the accumulation of several transient oscillation around the same frequency. Therefore, the time and frequency of the oscillation can be determined by local peaks. Since the local peaks in these local-oscillation TFRs have a linear power ratio [23], the Equation (5) needs to be converted to linear space.

(7) $$R_{\mathrm{mix}}\left(t,f\right)=R_{p}\left(t,f\right)R_{\mathrm{ap}}\left(t,f\right)$$

An aperiodic component in linear space can be expressed as

(8) $$R_{\mathrm{ap}}\left(t,f\right)=10^{E_{\mathrm{ap}}\left(t,f\right)}=\frac{10^{b_{t}}}{f^{\chi_{t}}}$$

Then, the TFR of oscillatory activities in linear space can be expressed as

(9) $$R_{p}\left(t,f\right)=\frac{R_{\mathrm{mix}}\left(t,f\right)}{R_{\mathrm{ap}}\left(t,f\right)}=\frac{f^{\chi_{t}}}{10^{b_{t}}}R_{\mathrm{mix}}\left(t,f\right)$$

The formula (9) shows that the peak power of oscillation returned by the proposed algorithm always refers to the aperiodic adjusted power [7,23]. This means that the power of oscillation TFR is defined as the magnitude of the peak over and above the aperiodic component in the original time–frequency spectrum. Therefore, we can obtain the TFR of oscillations from an aperiodic background. Then, we can parameterize oscillations in specific frequency bands based on $R_{p}\left(t,f\right)$.

#### 2.3.3. Oscillation Detection

In this paper, the detected oscillations are defined as transient events in the single-trial oscillation TFR [15,23]. Here, the corresponding time–frequency values of the local maximum are taken as the peak frequency and peak time of a single local oscillation event. Duration and frequency span of local oscillation are calculated with full width at half maxima (FWHM) (see Figure S1B in the Supplement).

### 2.4. Compared with Existing Methods

In order to better illustrate the performance of the proposed algorithm, we tried to compare our algorithm with previously reliable algorithms for typical time–frequency parameterization of oscillatory activities. Based on this requirement, we compared the performance of our algorithm with the following two typical methods: (1) SPRiNT [26] and (2) s-PAPTO, which was adapted from the PATPTO [23] by replacing the wavelet transform with SLT in this paper. In our simulations, both algorithms were used according to their standard processes. The implementation of SPRiNT was based on the code provided at https://github.com/lucwilson/SPRiNT (accessed on 15 June 2023). The implementation of s-PAPTO was based on the code provided at https://github.com/tbardouille/papto_camcan (accessed on 10 July 2023).

#### The Evaluation of the Performance of the Proposed Algorithm

- The mean absolute error (MAE) of oscillation parameters

Because peak frequency, frequency span, onset time, and duration can reflect the fluctuations in most features of neural oscillatory activities. We defined the errors of four oscillation parameters calculated by algorithms as the absolute differences between these parameters and the ground values. To evaluate the performance of the three algorithms, the MAEs of four oscillation parameters based on the three algorithms were calculated, which were defined as the mean of absolute errors of those parameters calculated by three algorithms calculated by three algorithms. The standard error of the mean (SEM) of errors was also calculated. 

Although the main parameters of oscillation are the peak frequency, frequency span, and onset time and duration, it may be necessary to select appropriate parameters of oscillatory activities in the practice data analysis (see Results).

- The coefficient of variation (CV) in multi-trial OPM-MEG oscillation parameters

Since the proposed method calculated the oscillation parameters for each trial in multi-trial OPM-MEG data, CV was introduced to evaluate the deviation of multi-trial oscillation parameters. A CV value below 1 indicates a low deviation [39], which means that the oscillation parameters are consistent between trials.

### 2.5. Statistical Methods

For comparing algorithms in simulations, SQUID-MEG and OPM-MEG are relevant. The Bayes factor came from a Bayesian independent samples *t*-test [40]. This operation was performed with JASP (Version 0.18.3) (https://jasp-stats.org/ (accessed on 20 March 2024). Welch’s *t*-test was used to compute the p-values. For all tests in simulation, the alternative hypothesis was that the parameter error of SPRiNT or s-PAPTO was greater than that of STPPTO. For all tests in the comparison of algorithms in SQUID-MEG and OPM-MEG, the alternative hypothesis was that the parameters of SPRiNT or s-PAPTO were not equal to that of STPPTO.

For SQUID-MEG and OPM-MEG, since the SQUID-MEG and OPM-MEG in this paper came from different numbers of groups, the Bayes factor was obtained with a Bayesian Mann-Whitney U test based on a data augmentation algorithm with 5 chains of 1000 iterations with JASP. The alternative hypothesis was that the parameters of OPM-MEG or were not equal to those of SQUID-MEG. For comparisons in tasked-related OPM-ME. Welch’s *t*-test was used to compute p-values. This statistical analysis was performed with Python. 

## 3. Results

Firstly, we validated the performance of STPPTO for parameterizing oscillations, accompanied by quantitative comparisons with statistical methods against existing algorithms (SPRiNT and s-PATPTO) in simulations. Secondly, we verified that there was less difference in oscillatory parameters calculated by STPPTO between resting-state SQUID-MEG and OPM-MEG, accompanied by quantitative comparisons against existing algorithms using Statistical methods. Thirdly, we applied STPPTO on FS OPM-MEG. We verified the high reliability of the oscillation parameters solved by STPPTO across trials by calculating the coefficient of variation (CV) for the frequency span, onset time, duration, and peak power of multi-trial FS OPM-MEG data. Finally, we applied STPPTO on the oscillation response between different-order harmonics and dominant cerebral hemispheres under FS for further analysis.

### 3.1. The Performance of STPPTO against Simulation

To investigate algorithm performance, we first simulated neural oscillation with known ground truth parameters combined with an aperiodic component. The ground truth parameters of oscillation included the peak frequency, frequency span, onset time, and duration. Subsequently, the measured noise was added to Simulation I to generate the signal for Simulation II. The absolute error of peak frequency, frequency span, onset time, duration, and peak time of the oscillations were used to evaluate the performance of the algorithms. Then, we used the Welch *t*-test to calculate the significance of differences (*p*) between the algorithms. To quantify the comparisons between algorithms, the Bayes factor (BF_10_) was obtained with a Bayesian independent samples *t*-test. 

In Simulation I, Table 1 shows the mean absolute error (MAE) and standard error of the mean (SEM) of the absolute error of oscillation parameters (peak frequency, frequency span, onset time, and duration) calculated by SPRiNT, s-PAPTO, and STPPTO. The result shows that the proposed method can calculate the peak frequency (SPRiNT vs. STPPTO: *BF*_10_ > 100. s-PAPTO vs. STPPTO: *BF*_10_, 119.21), frequency span (s-PAPTO vs. STPPTO: *BF*_10_ > 100), onset time (SPRiNT vs. STPPTO: *BF*_10_ > 100. s-PAPTO vs. STPPTO: *BF*_10_ > 100), and duration (s-PAPTO vs. STPPTO: *BF*_10_, 0.17) with smaller errors than other algorithms. The error of the peak frequency, frequency span, onset time, and duration of the proposed method can be kept at 0.75 ± 0.02 Hz, 1.43 ± 0.05 Hz, 0.101 ± 0.002 s, and 0.100 ± 0.003 s, respectively.

**Figure 2:**
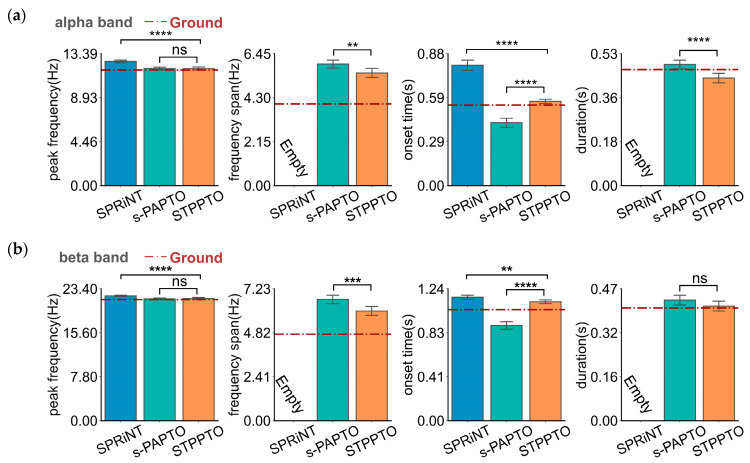
The parameters of alpha-band (**a**) and beta-band (**b**) oscillations calculated by SPRiNT, s-PAPTO, and STPPTO in Simulation II. Ground is the ground truth of oscillation parameters. Error bars represent a 95% confidence interval (C.I.). **** *p* < 0.0001, *** *p* < 0.001, ** *p* < 0.01, * *p* < 0.05, and ns *p* > 0.05.

Even under noisy conditions (in Simulation II), STPPTO can obtain oscillatory parameters closer to ground values than SPRiNT and s-PAPTO (in Figure 2a). Furthermore, Table 2 shows the mean absolute error (MAE) and standard error of the mean of absolute error (SEM) of peak frequency, frequency span, onset time, and duration calculated by SPRiNT, s-PAPTO, and STPPTO. It is evident that the proposed method can calculate peak frequency (SPRiNT vs. STPPTO: *BF*_10_ > 100. s-PAPTO vs. STPPTO: *BF*_10_, 23.98), frequency span (s-PAPTO vs. STPPTO: *BF*_10_, 202.12), onset time (SPRiNT vs. STPPTO: *BF*_10_ > 100. s-PAPTO vs. STPPTO: *BF*_10_ > 100), and duration (s-PAPTO vs. STPPTO: *BF*_10_, 0.13) with less error. The error of the peak frequency, frequency span, onset time, and duration calculated by the proposed method can reach 1.19 ± 0.02 Hz, 2.61 ± 0.06 Hz, 0.151 ± 0.002 s, and 0.231 ± 0.005 s in noisy data, respectively. Overall, STPPTO can better parameterize oscillations than SPRiNT and s-PAPTO, whether considering the effect of the aperiodic component alone or in conjunction with the measured noise.

### 3.2. The Application of STPPTO

#### 3.2.1. The Transient Oscillation in the Primary Visual Cortex under a Resting State

**Figure 3:**
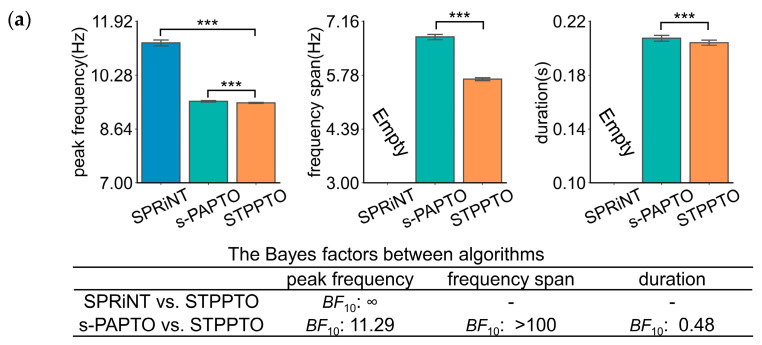

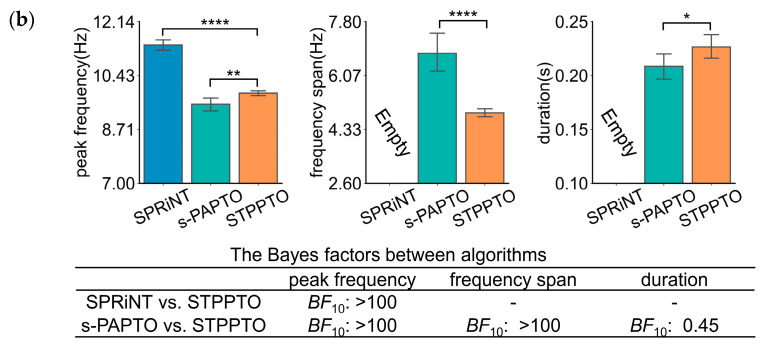
The results of the transient alpha oscillations of the primary visual cortex in resting-state SQUID-MEG (**a**) and OPM-MEG (**b**). Top panel: the mean bar charts of transient alpha oscillation peak frequency, frequency span, and duration with SPRiNT, s-PAPTO, and STPPTO. Error bars represent 95% C.I. for all transient oscillations of all subjects. **** *p* < 0.0001, *** *p* < 0.001, ** *p* < 0.01, and * *p* < 0.05. Bottom panel: Bayes factor quantifying peak frequency, frequency span, and duration of STPPTO compared to those of SPRiNT and s-PAPTO. This information of the beta band is shown in Figure S3.

This section applies STPPTO to parameterize oscillations in specific frequency bands of ROIs in resting-state SQUID-MEG and OPM-MEG. The results were accompanied by comparisons against those of SPRiNT and s-PAPTO. For the resting state, the onset time of these transient oscillations was not taken into consideration. Firstly, peak frequency, frequency span, and duration of the alpha and theta bands were obtained using three algorithms. *p*-values and Bayes factors were calculated between STPPTO and other algorithms with statistical methods. The peak frequency and frequency span calculated by different methods are significantly different for both alpha (Figure 3) and beta bands (Figure S3). For SQUID-MEG, Figure 3a shows a higher difference between SPRiNT and STPPTO (SPRiNT vs. STPPTO: *BF*_10_, ∞) than s-PAPTO and STPPTO (s-PAPTO vs. STPPTO: *BF*_10_, 11.29) on peak frequency. There was a strong difference between s-PAPTO and STPPTO (s-PAPTO vs. STPPTO: *BF*_10_ > 100) on the frequency span. There was a slight difference between s-PAPTO and STPPTO (s-PAPTO vs. STPPTO: *BF*_10_, 0.48) on duration. For OPM-MEG, similarly, Figure 3b shows a higher difference between SPRiNT and STPPTO (SPRiNT vs. STPPTO: *BF*_10_ > 100) than s-PAPTO and STPPTO (s-PAPTO vs. STPPTO: *BF*_10_ > 100) on peak frequency. There was also a strong difference between s-PAPTO and STPPTO (s-PAPTO vs. STPPTO: *BF*_10_ > 100) on the frequency span. There was also a slight difference between s-PAPTO and STPPTO (s-PAPTO vs. STPPTO: *BF*_10_, 0.45) on duration. 

Due to the number of participants and experimental environment, there were differences in peak frequency, frequency span, duration, and peak power between transient oscillations between SQUID-MEG and OPM-MEG, as shown in Table 3. But, the difference in peak frequency was less than 5% and the Bayes factor was less than 1 (SQUID-MEG vs. OPM-MEG, *BF*_10_: 0.52 for alpha band, 0.37 for beta band). It means that there is little difference between the transient oscillations recorded by SQUID-MEG (9.44 ± 0.01 Hz for alpha band, 19.69 ± 0.02 for beta band) and OPM-MEG (9.85 ± 0.04 for alpha band, 19.84 ± 0.08 for beta band) on peak frequency. Similarly, there is little difference (SQUID-MEG vs. OPM-MEG, *BF*_10_: 0.24 for alpha band, 0.29 for beta band) between SQUID-MEG (0.204 ± 0.001 s for alpha band, 0.181 s for beta band) and OPM-MEG (0.227 ± 0.005 s for alpha band, 0.205 ± 0.005 s for beta band) on oscillation duration. The result demonstrated that there was a small difference between OPM-MEG and SQUID-MEG in recording oscillation information, which demonstrated that OPM-MEG can record the transient oscillations reliably as SQUID-MEG can do. And, it described the frequency and duration of alpha and beta transient oscillations in the primary visual cortex. But there were significant differences in frequency span (SQUID-MEG vs. OPM-MEG, *BF*_10_: 41.97 for the alpha band, 66.34 for the beta band) and peak power (SQUID-MEG vs. OPM-MEG, *BF*_10_: 16.57 for the alpha band, 884.25 for the beta band) between OPM-MEG and SQUID-MEG. 

#### 3.2.2. The Oscillations in the Visual Cortex under Rhythmic Flash Stimulus OPM-MEG

Neural oscillation studies on rhythmic stimuli have reached a general conclusion. It has been summarized in previous studies [37,38,39]. Therefore, this phenomenon will not be reiterated here but will mainly be used to elucidate other laws and phenomena calculated by STPPTO.

**Figure 4:**
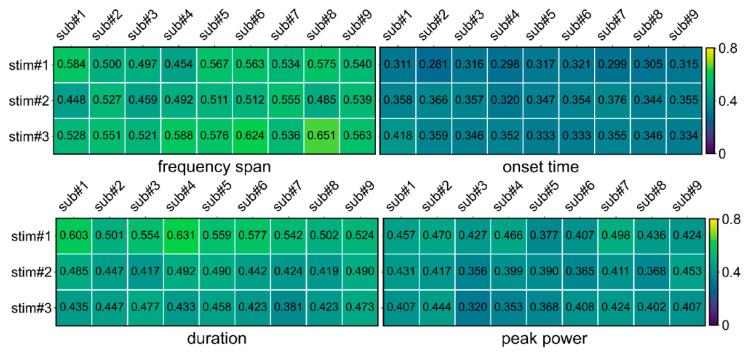
The CVs of frequency span, onset time, duration, and peak power for multiple trials. stim#1, stim#2, and stim#3 represent the 3 Hz, 6.5 Hz, and 8.5 Hz stimuli, respectively.

Firstly, multi-trial CVs of frequency span, onset time, duration, and peak power were calculated for multiple trials of multiple subjects under three FS stimuli. As shown in Figure 4, most of the CVs are less than 0.6, and the mean (SEM) CV of all of the parameters was 0.442. It indicates that the parameters solved by STPPTO had a small deviation between trials and had high reliability.

**Figure 5:**
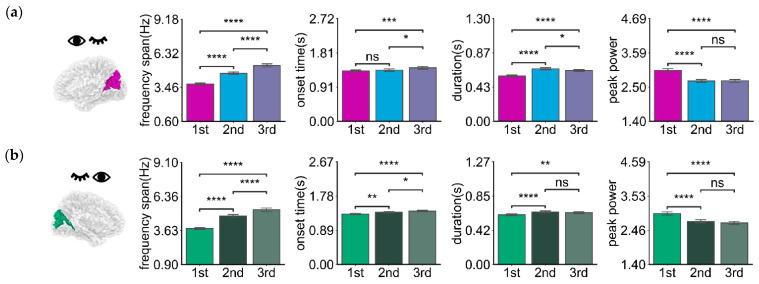
The mean bar charts of frequency span, onset time, duration, and peak power of oscillation of the dominant hemisphere with 1st, 2nd, and 3rd order harmonic of the left-eye (**a**) (ROIs: Right V1 + O6) and right-eye (**b**) (ROIs: Left V1 + O6) stimulus. Error bars represent 95% C.I. **** *p* < 0.0001, *** *p* < 0.001, ** *p* < 0.01, * *p* < 0.05, and ns *p* > 0.05.

**Figure 6:**
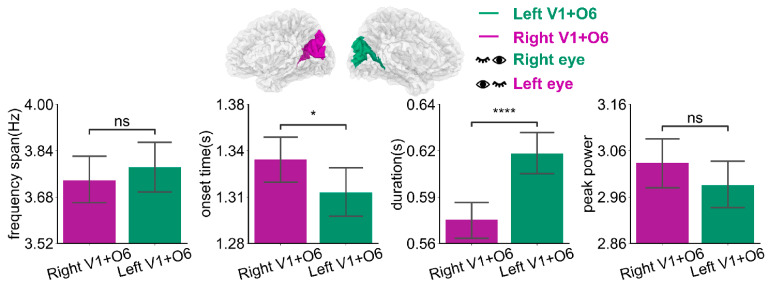
The mean bar of frequency span, onset time, duration, and peak power of oscillations in the primary visual cortex of the different dominant hemispheres. Error bars represent 95% C.I. **** *p* < 0.0001, * *p* < 0.05, ns *p* > 0.05.

Oscillatory activities in the right dominant hemisphere Right V1 + O6 under left-eye stimulation are depicted in Figure 5a and Table 4. The frequency span of the first oscillations was smaller than that of the second and third orders (*p* < 0.0001). The onset time (after stimulus onset) of the first oscillations was earlier than those of the second and third order (*p* < 0.05, *p* < 0.001, respectively). The duration of the first oscillations was slightly shorter than that of the second and third order (*p* < 0.0001). The peak power of the first oscillations was slightly lower than that of the second and third order (*p* < 0.0001). For the left dominant hemisphere Left V1 + O6 under right eye stimulation, as shown in Figure 5b and Table 4, the frequency span of the oscillations is smaller than that of the second and third order (*p* < 0.0001). The onset time (after stimulus onset) of the first oscillations was earlier than those of the second and third order (*p* < 0.01, *p* < 0.0001, respectively). The duration was slightly shorter than that of the second and third order (*p* < 0.0001 and *p* < 0.01, respectively). The peak power of the first oscillations was slightly lower than that of the second and third order (*p* < 0.0001). The first oscillation occurred earlier than the second and third order and its duration was slightly shorter than that of the second and third order. At the same time, the oscillation response varies for different dominant hemispheres, as Figure 6 and Table 5 show. There was a significant difference in onset time (*p* < 0.05) and duration (*p* < 0.0001) between different dominant hemispheres under unilateral stimulation. The oscillatory activities in the left dominant hemisphere occurred earlier (approximately 25 ms, *p* < 0.05) and lasted longer (about 40 ms, *p* < 0.0001) than those in the right dominant hemisphere. There was a slight difference in values for frequency span and peak power (*p* > 0.05).

## 4. Discussion

The analysis of neural oscillation holds a significant place in neuroscience. Researchers are interested in analyzing the relationship between actual physiological activities and oscillation parameters such as frequency, time, duration, and power. In this paper, we propose STPPTO, a new analysis algorithm for oscillations in specific frequency bands. It adopts SLT with high resolution [27] to calculate the time–frequency spectrum. Meanwhile, it introduces a time-resolved manner to fit aperiodic components at each time point, and removes these components, thereby reducing the influence of time-varying aperiodic components on parameterizing oscillations. Then, the transient event definition (based on local maximum and full width at half maximum) is used to calculate the parameters of the (transient) oscillations. We used simulated neural data to illustrate the performance of the method compared with PAPTO [23] and SPRiNT [26]. In the simulation, the proposed method outperformed other methods in calculating oscillation parameters, even when noise is present (Figure 2, Table 1 and Table 2). The *BF*_10_ value for the onset time calculation error between the proposed algorithm and other algorithms was remarkably high, suggesting that the error of the proposed method is significantly smaller than that of other methods. This is partly due to the super-resolution of SLT and partly due to the time-resolved manner.

We also verify a small difference in oscillation parameters calculated by the proposed method in the resting-state primary visual cortex region recorded by SQUID-MEG and OPM-MEG. Since OPM-MEG is a relatively new MEG technology, studies on SQUID-MEG and OPM-MEG under the same paradigms focus on verifying the effectiveness and performance of OPM-MEG, including signal recording [41,42] and source localization [43,44]. However, some researchers have turned to oscillation analysis [5]. In this paper, we found a slight difference in parameters of the alpha and beta band oscillations of the primary visual cortex between the two techniques, particularly on peak frequency and duration. The values of peak frequency and oscillation duration were similar to the results of previous studies [7,45]. However, the peak power of OPM-MEG was significantly greater than that of SQUID-MEG (*BF*_10_ > 100 for beta transient oscillation), which may be attributed to the higher sensitivity of OPM sensors to changes in MEG [46,47]. At the same time, we quantified the comparison of different algorithms for parameterizing transient oscillations in SQUID-MEG and OPM-MEG. We found some similar differences to the simulations, which further illustrated the difference in performance between algorithms. 

In the analysis of a multi-trial task, we used CVs to evaluate the deviation of multi-trial parameters. The lower CVs verify the consistency of the proposed method in solving the oscillation parameters in OPM-MEG. In a further extended application of STPPTO, we analyzed the relationship between time–frequency parameters of different-order harmonics (Figure 5). Moreover, an analysis was also conducted on the oscillation of dominant hemispheres (Figure 6). These results are only preliminary summaries of the oscillation response to rhythmic stimuli. However, the time–frequency information provides more detailed insights that have not been included in most previous studies on oscillations in response to rhythmic stimuli [5,48]. For example, we demonstrated that the oscillation in the left dominant hemisphere occurs earlier (*p* < 0.05) and lasts longer (*p* < 0.0001) than that of the right dominant hemisphere. The enhanced parameter-capturing accuracy of STPPTO further revealed the differences in different-order harmonic oscillations under rhythmic stimulation, as well as the differences between dominant hemispheres.

Overall, STPPTO is a new method for time–frequency parameterizing specific-frequency-band oscillations from a time-varying aperiodic background. Our results showed that STPPTO can better capture the time–frequency parameters of oscillatory activities compared to traditional algorithms. The proposed method is suitable for scenarios where there are transient oscillations in resting or task-related situations. It is well known that task states are accompanied by changes in transient activity. However, most studies just focus on the parameters of static spectra, such as peak frequency, bandwidth, and power, as neural activity remains steady during the resting state. These parameters can reflect the speed and power of oscillations, which are related to the brain’s physiological status, such as age [7,19,49], and disease [21,50]. For the purpose of analyzing these features of oscillations, parameterization based on static spectra [7,51] is sufficient. Additionally, there are bursts of transient oscillatory activity in the resting state [52]. The duration and frequency of these transient oscillations are related to physiological states such as age [23] but they have not been widely analyzed. Therefore, we can choose between a static spectral parameterization or a transient oscillatory parameterization, depending on the type and requirements of the data to be analyzed. With subsequent refinement of the algorithm, it will provide a new analysis method for data-driven neurodynamic research.

- Limitations of the study

Due to the 1/f nature of the aperiodic background power distribution, it is particularly important to distinguish the fluctuations in low-frequency oscillations (including mu, theta, and delta events) from aperiodic activities. Although this paper focuses on the importance of separating most oscillatory activity from the aperiodic background, the low-frequency oscillation shows greater variation when the aperiodic component is removed using the proposed method in this study. Low-frequency oscillation (<4 Hz) reflects the neurophysiological mechanisms of the brain [53,54]. Otherwise, the proposed method may discard transient impulse responses, which generate a valid and broad spectrum and reflect some brain dynamics [55,56]. Therefore, studies on low-frequency oscillations and transient impulse response are needed to clarify the extent of these changes and their potential impacts in the future.

Additionally, in the SQUID-MEG dataset and the OPM-MEG experiments described in this paper, participants were instructed to keep their eyes open, which is consistent with prior research. However, it is well-documented that the eyes-open and eyes-closed states can induce notable differences in brain oscillatory activity, particularly within the alpha band [57,58]. These differences could lead to disparate results of transient oscillations during resting states. Therefore, our future studies will analyze and compare transient oscillatory activity in the resting brain under both conditions.

Although the proposed algorithm can effectively capture the oscillation activity parameters, there is potential to utilize this information to obtain inter-region correlation or spatial-time–frequency fluctuations in oscillations [39,59] in the future. In addition, the differences in peak power between multi-order harmonics in this paper are similar to those of most of the previous steady-state evoked experiments [48,60]. However, the other parameters, such as onset time, duration, and so on, require a large amount of data to verify in the future. The impact of these oscillation parameters on other brain activities is not covered in this paper, such as sensory time perception and high-order cognition [61]. Therefore, applications of the proposed algorithm in more complex MEG experiments are also an important part of its development.

## 5. Conclusions

STPPTO is a new neural oscillation time–frequency parameterization method from a time-varying aperiodic background. The results of simulations verified that the algorithm had better performance on oscillation time–frequency parameterization. The results of MEG experiments further verified the effectiveness of the algorithm. Our results showed that STPPTO can better capture the time–frequency parameters of oscillatory activities compared with traditional algorithms. The improved parameter-capturing accuracy of STPPTO further revealed the differences in the harmonic oscillation of different orders under rhythmic stimulation, as well as the differences between dominant hemispheres. With the subsequent refinement of the algorithm, it will provide a new analysis method for data-driven neurodynamic research.

## Figures and Tables

**Table 1:** The errors (MAE ± SEM) of peak frequency, frequency span, onset time, duration, and peak time of SPRiNT, s-PAPTO, and STPPTO in Simulation I, along with the *p*-values and Bayes factors for the comparison between algorithms.

	Peak Frequency Error (Hz)	Frequency Span Error (Hz)	Onset Time Error (s)	Duration Error (s)
SPRiNT [26]	1.48 ± 0.03	-	0.315 ± 0.008	-
s-PAPTO [23]	0.88 ± 0.02	1.89 ± 0.06	0.206 ± 0.007	0.105 ± 0.003
STPPTO	0.75 ± 0.02	1.43 ± 0.05	0.100 ± 0.003	0.101 ± 0.002
SPRiNT [26] vs. STPPTO	*p* < 0.0001	-	*p* < 0.0001	-
	*BF*_10_: >100	-	*BF*_10_: >100	-
s-PAPTO [23] vs. STPPTO	*p* < 0.0001	*p* < 0.0001	*p* < 0.01	ns.
	*BF*_10_: >100	*BF*_10_: >100	*BF*_10_: >100	*BF*_10_: 0.17

**Table 2:** The errors (MAE ± SEM) of peak frequency, frequency span, onset time, duration, and peak time of SPRiNT, s-PAPTO, and STPPTO in Simulation II, along with the p-values and Bayes factors for the comparison between algorithms.

	Peak Frequency Error (Hz)	Frequency Span Error (Hz)	Onset Time Error (s)	Duration Error (s)
SPRiNT [26]	1.40 ± 0.03	-	0.314 ± 0.008	-
s-PAPTO [23]	1.39 ± 0.03	3.20 ± 0.07	0.399 ± 0.008	0.165 ± 0.004
STPPTO	1.19 ± 0.02	2.61 ± 0.06	0.231 ± 0.005	0.151 ± 0.002
SPRiNT [26] vs. STPPTO	*p* < 0.0001	-	*p* < 0.0001	-
	*BF*_10_: >100	-	*BF*_10_: >100	-
s-PAPTO [23] vs. STPPTO	*p* < 0.0001	*p* < 0.0001	*p* < 0.0001	*p* < 0.01
	*BF*_10_: 23.98	*BF*_10_: >100	*BF*_10_: >100	*BF*_10_: 0.13

**Table 3:** The mean ± SEM and difference (*difference* and *BF*_10_) of peak frequency, frequency span, duration, and peak power of transient alpha and beta oscillations of SQUID-MEG and OPM-MEG based on STPPTO.

		Peak Frequency (Hz)	Frequency Span (Hz)	Duration (s)	Peak Power
alpha	SQUID-MEG	9.44 ± 0.01	5.67 ± 0.01	0.204 ± 0.001	3.63 ± 0.02
	OPM-MEG	9.85 ± 0.04	4.82 ± 0.07	0.227 ± 0.005	4.36 ± 0.04
	*BF*_10_	0.52	41.97	0.24	16.57
	*Difference*	4%	15%	9%	19%
beta	SQUID-MEG	19.69 ± 0.02	6.86 ± 0.02	0.181 ± 0	3.15 ± 0
	OPM-MEG	19.84 ± 0.08	5.54 ± 0.06	0.205 ± 0.005	3.91 ± 0.03
	*BF*_10_	0.37	66.34	0.29	>100
	*Difference*	1%	19%	13%	24%

$\mathrm{Difference}=\frac{\mathrm{parameter}\;\mathrm{difference}\;\mathrm{between}\;\mathrm{SQUID}-\mathrm{MEG}\;\mathrm{and}\;\mathrm{OPM}-\mathrm{MEG}}{\mathrm{parameter}\;\mathrm{value}\;\mathrm{of}\;\mathrm{SQUID}-\mathrm{MEG}}.$

**Table 4:** The values (mean ± SEM) of frequency span, onset time, duration, and peak power of 1st, 2nd, and 3rd order harmonic oscillation response of the dominant hemisphere.

ROI	Harmonic Order	Frequency Span (Hz)	Onset Time(s)	Duration	Peak Power
Right V1 + O6	1st	3.74 ± 0.04	1.338 ± 0.009	0.574 ± 0.006	3.03 ± 0.03
	2nd	4.63 ± 0.06	1.368 ± 0.010	0.665 ± 0.007	2.69 ± 0.02
	3rd	5.30 ± 0.07	1.386 ± 0.105	0.644 ± 0.006	2.70 ± 0.02
Left V1 + O6	1st	3.78 ± 0.04	1.313 ± 0.009	0.614 ± 0.006	2.99 ± 0.03
	2nd	4.80 ± 0.06	1.359 ± 0.011	0.650 ± 0.006	2.75 ± 0.03
	3rd	5.30 ± 0.07	1.389 ± 0.011	0.640 ± 0.006	2.70 ± 0.02

**Table 5:** The values (mean ± SEM) of frequency span, onset time, duration, and peak power of oscillations of the primary visual cortex of the different dominant hemispheres.

Stimulus-ROI	Frequency Span (Hz)	Onset Time (s)	Duration	Peak Power
Right eye-Left V1 + O6	3.74 ± 0.04	1.338 ± 0.009	0.574 ± 0.006	3.03 ± 0.03
Left eye-RightV1 + O6	3.78 ± 0.04	1.313 ± 0.009	0.614 ± 0.006	2.99 ± 0.03

## Data Availability

The SQUID-MEG dataset analyzed in this paper is available in the Donders repository [MOUS dataset, https://data.donders.ru.nl/collections/di/dccn/DSC_3011020.09_236?1 (accessed on 20 December 2023). Schoffelen, J.M., Oostenveld, R., Nietzsche Lam, Julia Udden, Annika Hultén, and Hagoort, P. (2019): Mother of unification studies, a 204-subject multimodal neuroimaging dataset to study language processing. Version 1. Radboud University. (dataset). https://doi.org/10.34973/37n0-yc51 (accessed on 20 December 2023). All OPM-MEG data reported in this paper will be shared by the lead contact upon request.

## Supplementary Materials

The following supporting information can be downloaded at: https://www.mdpi.com/article/10.3390/bioengineering11080773/s1, Figure S1: The alogrithm of STPPTO including seperating aperodic component; Figure S2. The oscillation power separete from aperiodic background with different method(errors); Figure S3. The result of transient alpha oscillations of primary visual cortex in resting-state SQUID-MEG; Table S1: Table S1 The paremeters of simulated data.
