# Supplementary material for: A Novel Time–Frequency Parameterization Method for Oscillations in Specific Frequency Bands and Its Application on OPM-MEG
Source: Bioengineering (Basel). 2024 Jul 31;11(8):773. doi: 10.3390/bioengineering11080773 (PMC11351447; doi:10.3390/bioengineering11080773)
Supplement: Supplementary file 1 [file bioengineering-11-00773-s001.zip › bioengineering-3078921-supplementary.pdf]

Appendix

Table S1 The paremeters of simulated data.

Peak frequency (Hz)	bandwidth (Hz)	onset time(s)	duration(s)
3	1	0.2	0.8
12	2	0.5	0.6
22	3	1.0	0.5
32	4	1.5	0.3

(a)

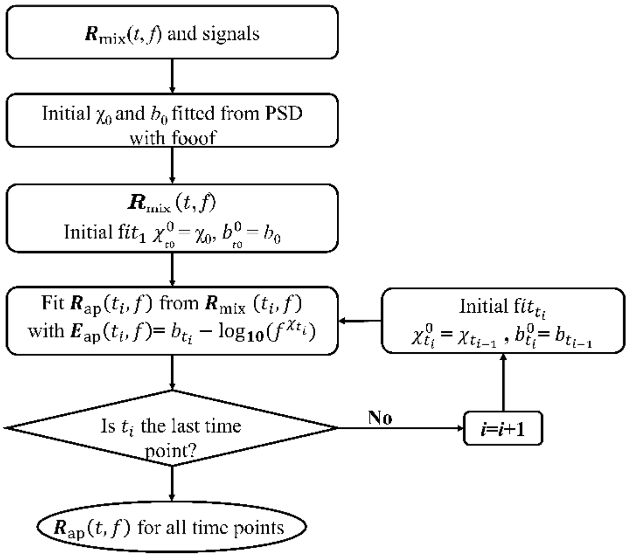

(b)

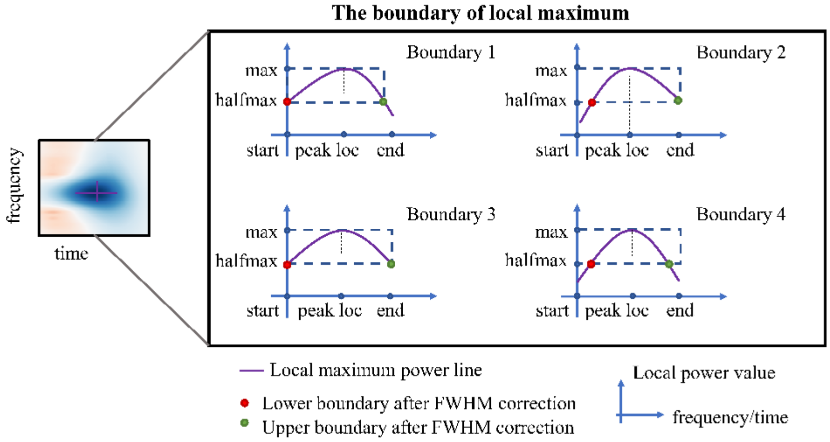

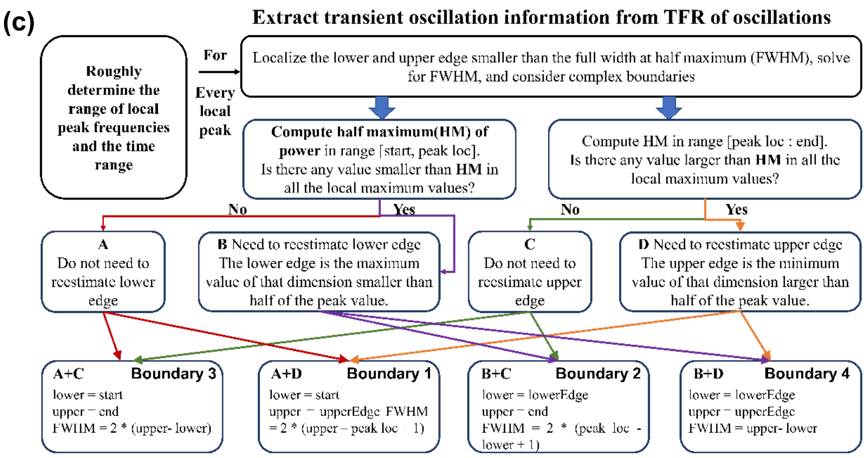

Figure S1. The alogrithm of STPPTO including seperating aperodic component(a) and computing the time-frequency boundary of transient oscillation based on local maximum (b and c).

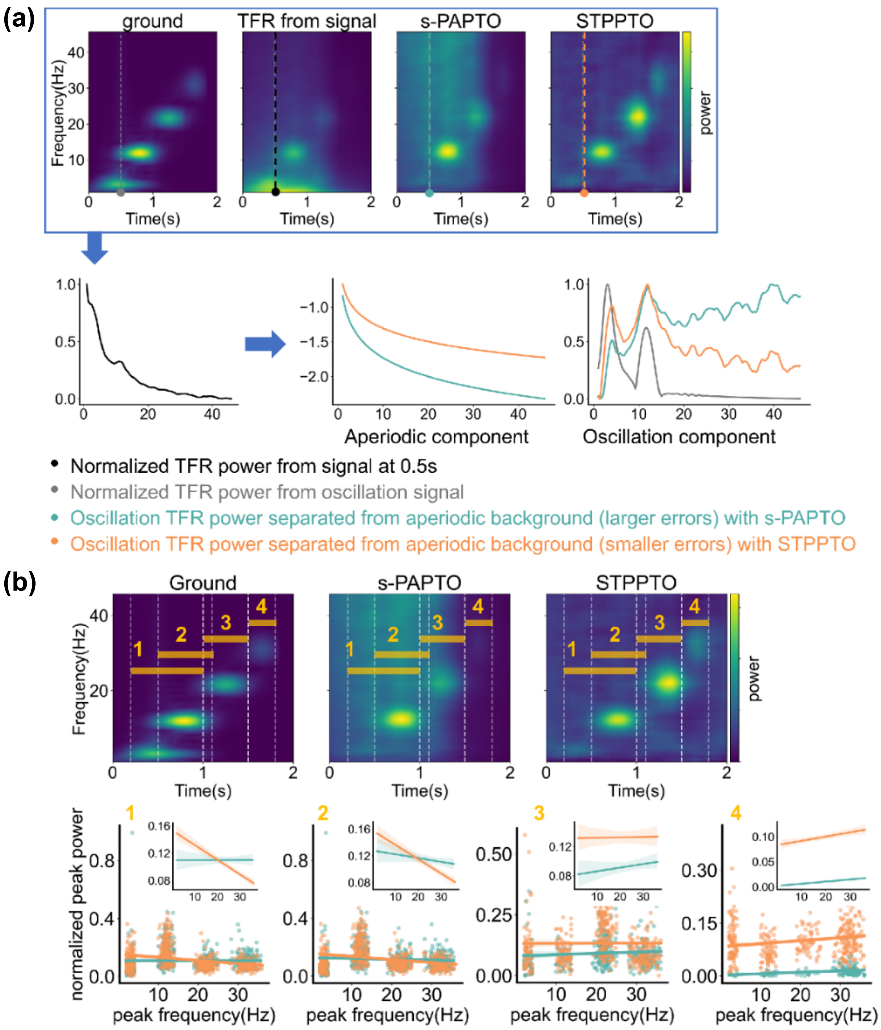

Figure S2. The oscillation power separete from aperiodic background with different method(errors). a. The oscillation power at 0.5s of ground oscillation TFR, mix TFR, oscillation TFR based on s-papto and STPPTO (in Simulation I). d. The relationship between peak frequency and normalized peak power of oscillation TFR based on s-papto and STPPTO at different peroids (in Simulation I).

(a)

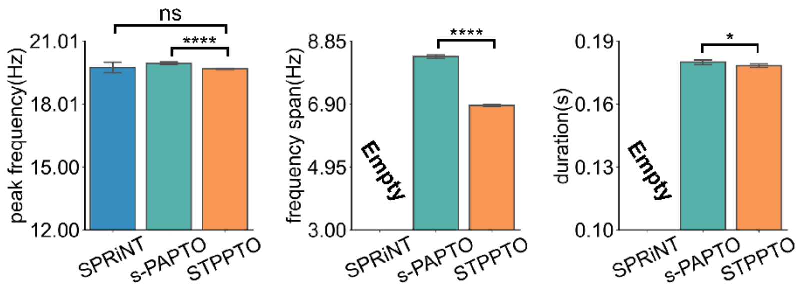

The Bayes factors between algorithms

	peak frequency	frequency span	duration
SPRiNT vs. STPPTO	BF_{10}: 0.05	-	-
s-PAPTO vs. STPPTO	BF_{10}: >100	BF_{10}: ∞	BF_{10}: 0.49

(b)

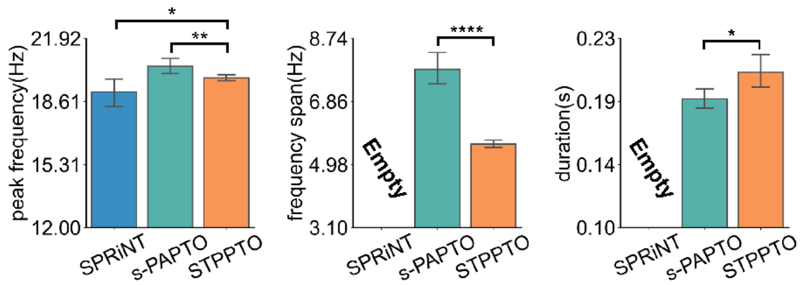

The Bayes factors between algorithms

	peak frequency	frequency span	duration
SPRiNT vs. STPPTO	BF_{10}: 3.26	-	-
s-PAPTO vs. STPPTO	BF_{10}: 13.82	BF_{10}: >100	BF_{10}: 0.71

Figure S3. The result of transient alpha oscillations of primary visual cortex in resting-state SQUID-MEG(a) and OPM-MEG(b). Top panel: the mean bar charts of transient alpha oscillation peak frequency, frequency span and duration with SPRiNT, s-PAPTO and STPPTO. Error bars represent 95% C.I. for all transient oscillations of all subjects. *** p<0.001, ** p<0.01, * p<0.05, ns p>0.05. Bottom panel: Bayes factor quantifying peak frequency, frequency span and duration of STPPTO compared to those of SPRiNT, s-PAPTO.
